# Longitudinal proteomic profiling reveals increased early inflammation and sustained apoptosis proteins in severe COVID-19

**DOI:** 10.1038/s41598-020-77525-w

**Published:** 2020-11-25

**Authors:** Liis Haljasmägi, Ahto Salumets, Anna Pauliina Rumm, Meeri Jürgenson, Ekaterina Krassohhina, Anu Remm, Hanna Sein, Lauri Kareinen, Olli Vapalahti, Tarja Sironen, Hedi Peterson, Lili Milani, Anu Tamm, Adrian Hayday, Kai Kisand, Pärt Peterson

**Affiliations:** 1 Molecular Pathology, Institute of Biomedicine and Translational Medicine, University of Tartu, Tartu, Estonia; 2 Institute of Computer Sciences, University of Tartu, Tartu, Estonia; 3 Department of Virology, Faculty of Medicine and Department of Veterinary Biosciences, University of Helsinki, Helsinki, Finland; 4 Institute of Genomics, University of Tartu, Tartu, Estonia; 5 United Laboratories, Tartu University Hospital, Tartu, Estonia; 6 Peter Gorer Department of Immunobiology, School of Immunology and Microbial Sciences, King’s College London, London, UK; 7 Francis Crick Institute, London, UK

**Keywords:** Viral infection, Molecular medicine, Cytokines, Chemokines

## Abstract

SARS-CoV-2 infection has a risk to develop into life-threatening COVID-19 disease. Whereas age, hypertension, and chronic inflammatory conditions are risk factors, underlying host factors and markers for disease severity, e.g. requiring intensive care unit (ICU) treatment, remain poorly defined. To this end, we longitudinally profiled blood inflammation markers, antibodies, and 101 plasma proteins of hospitalized COVID-19 patients who did or did not require ICU admission. While essentially all patients displayed SARS-CoV-2-specific antibodies and virus-neutralization capacity within 12–15 days, a rapid, mostly transient upregulation of selective inflammatory markers including IL-6, CXCL10, CXCL11, IFNγ, IL-10, and monocyte-attracting CCL2, CCL7 and CCL8, was particularly evident in ICU patients. In addition, there was consistent and sustained upregulation of apoptosis-associated proteins CASP8, TNFSF14, HGF, and TGFB1, with HGF discriminating between ICU and non-ICU cohorts. Thus, COVID-19 is associated with a selective inflammatory milieu within which the apoptotic pathway is a cardinal feature with potential to aid risk-based patient stratification.

## Introduction

The coronavirus, SARS-CoV-2, has rapidly evolved into a global pandemic crisis. Although most infected individuals are either asymptomatic or develop mild symptoms, the infection poses the risk of potentially life-threatening COVID-19 disease to ~ 5–20% of symptomatic individuals, depending on their age group^1,2^.

A relatively rapid deterioration of lung function observed in many admitted to intensive care unit (ICU), has been commonly attributed to an overreacting immune response. However, other studies have questioned the generality of increases in a broad spectrum of cytokines or the association of virus-load with disease-severity^3^. Hence, there is a pressing need for greater granularity in characterizing the COVID-19 immune response, in which regard data currently available are highly informative but yet incomplete.

Thus, most patients across a range of disease severities were reported to display a core peripheral blood immune signature, in essence comprising hyperactivation and severe depletion of selective CD4^+^ and CD8^+^ αβ T cell subsets, Vγ9 Vδ2 T cells, and NK cells; adaptive B cell responses; and profound alterations in the composition of the blood monocyte and dendritic cell compartments^4–6^. Within this signature, discrete components were associated with disease-severity. Moreover, many but not all patients developed highly increased neutrophil-to-lymphocyte ratios, particularly as clinical manifestations diversify^7^.

Additionally, aberrant leukocyte counts and composition were associated with increases in discrete inflammatory markers, specifically C-reactive protein (CRP) and procalcitonin (PCT), while interleukin-6 (IL-6) has emerged as a biomarker for COVID-19 disease-course, with gradual but stark elevations associated with increased risk of death^8–11^. Likewise, proteomic profiling approaches and targeted analysis of plasma proteins have shown high IL-6, IL-10, IL-8 and IFNγ levels in many COVID-19 patients^5,6,10,12–15^.

Most patients reportedly develop antibodies to nucleocapsid (N) and spike (S) proteins^16–18^, including to RBD, a receptor-binding domain of the S protein, that is essential for virus entry into host cells via the ACE2 receptor^19^. Antibodies to the N protein, which protects viral RNA, are apparently prevalent in patients with SARS-CoV and SARS-CoV-2 infections^20,21^, and can comprise a relatively sensitive early biomarker for infection^22,23^.

With these findings as a backdrop, our study has undertaken a deep analysis of plasma in COVID-19 patients requiring ICU admission *versus* those who did not. We have mapped out longitudinal trajectories of antibodies and over 100 discrete plasma proteins, identifying traits particularly penetrant in the ICU cohort. Besides antibody formation and transient elevations of discrete cytokines, those traits conspicuously included a sustained, apoptosis-associated signature that composed a cardinal feature of disease, possibly related to the presentation of stark lymphopenia particularly in severely affected individuals.

## Results

We studied longitudinal plasma samples from 40 hospitalized COVID-19 patients, relative to declared onset of symptoms (Table 1). Because the patients were not treated with immunomodulatory drugs such as dexamethasone or IL-6 inhibitors during their hospitalization, therefore, the results mostly reflect the natural course of their disease. Based on oxygen levels and ICU requirements, the disease-status of 25 patients was classified as moderate and they were treated in the hospital ward (hereafter non-ICU). Conversely, 15 patients were classified as severe and were treated in the ICU (Supplementary Fig. 1A). Blood plasma samples were collected from most patients on almost every day of hospitalization. As the mean age of COVID-19 patients was 66 years (range 21–92 years), we compared their results to negative controls mostly comprising individuals over 60 years old. In some instances, we additionally studied a small group of SARS-CoV-2^+^ mild COVID-19 patients who attended the emergency medical department, but who were not hospitalized.

**Table 1:** Main characteristics of the patients and their comorbidities.

	All patients	ICU	Non-ICU
Number n	40	15	25
Mean age (age range) in years	66 (21–92)	66 (42–92)	65 (21–92)
Sex male/female %	45%(M)/55%(F)	60%(M)/40%(F)	36%(M)/64%(F)
Mean # days of admission to the hospital after the disease onset	8	9	8
Mean length of hospital stay in days (range)	18 (1–80)	27 (4–80)	12 (1–46)
Mean length of ICU stay	18	18	0
n and % with coronary diseases	29 (73%)	11 (73%)	18 (72%)
n and % with hypertension	21 (53%)	7 (47%)	14 (56%)
n and % with type 2 diabetes	8 (20%)	2 (13%)	6 (24%)
n and % with obesity	2 (5%)	2 (13%)	0
n and % with ARDS	6 (15%)	6 (40%)	0
n and % with respiratory insufficiency	13 (33%)	11 (73%)	2 (8%)
n and % with tumor	6 (15%)	3 (20%)	3 (12%)
n and % with kidney insufficiency	5 (13%)	3 (20%)	2 (8%)

### Blood inflammation markers and cell proportions

Compared to healthy control levels (< 5 mg/L), the CRP levels of ICU patients were highly elevated upon hospital admission (mean ~ 100 mg/L), and this difference continued during the disease course (peak mean value of ~ 225 mg/L, versus ~ 95 mg/L for non-ICU) (Supplementary Fig. 1B). The PCT levels were more comparable across the ICU/non-ICU cohorts at admission, but they too reached higher levels among ICU patients during their hospitalization (Supplementary Fig. 1C). Consistent with other reports, ICU patients had markedly decreased lymphocyte representation, and increased neutrophil representation (Supplementary Fig. 1D-G). Both the ICU and non-ICU cohorts displayed comparable and decreased blood basophil (Supplementary Fig. 1H) and eosinophil (Supplementary Fig. 1I) frequencies upon hospitalization, and those low levels were decreased for basophils over the disease course.

### Antibody trajectories to S1, S2, RBD and N proteins

**Figure 1:**
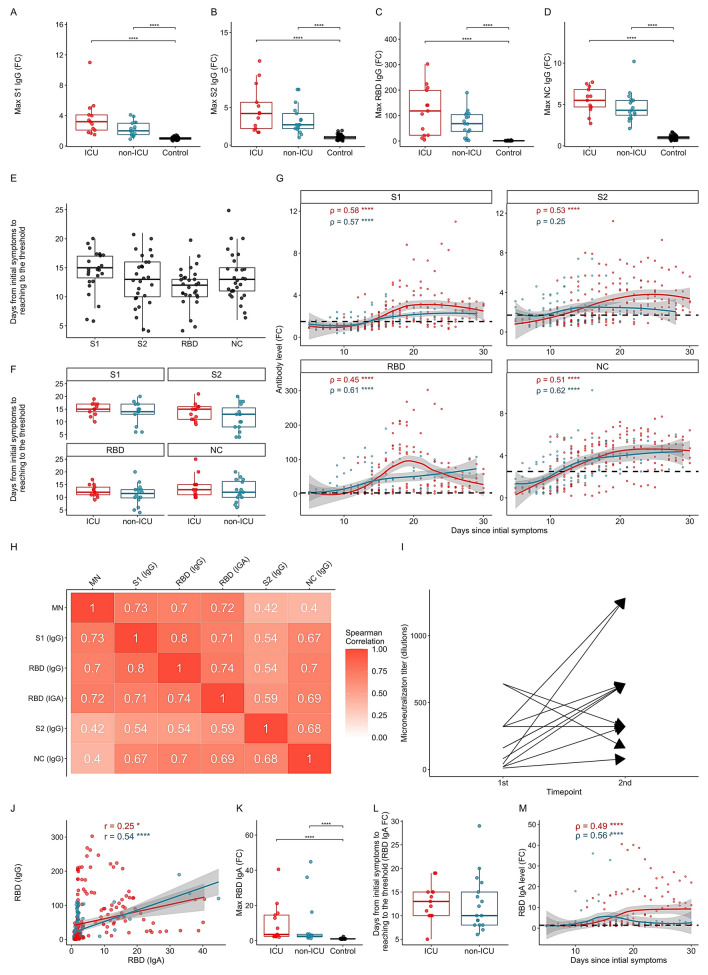
The trajectories of SARS-COV-2 antibodies. (**A**–**D**) Peak levels of IgG antibodies against SARS-CoV-2 spike protein subunits (**A**) S1, (**B**) S2, (**C**) its receptor binding domain (RBD) and (**D**) nucleocapsid protein (NC). Antibody levels are shown as fold changes (FC) over the average of healthy control samples, and the levels exceeding the arbitrary cutoff (+ 2SD of the average of negative controls) are shown as positive. (**E**) The time of the seroconversion of antibodies to S1, S2, RBD and N proteins in days since the start of the symptoms to the cutoff level. The seroconversion of RBD specific antibodies occurs first (median = 12, 0.25 quantile = 10, 0.75 quantile 13) compared to anti-S1 (15, 13.25–17), anti-S2 (13, 10–16) and anti-N (13 (11–15). (**F**) The seroconversion of antibodies between ICU and non-ICU groups. (**G**) Accumulated trajectory of individual antibody levels to four antigens plotted over the duration of the disease in ICU and non-ICU groups. The thresholds are shown as dashed black line. The antibody levels are shown as fold changes (FC) as in panels A-D. (**H**) Spearman’s correlations matrix between anti-SARS-CoV-2 antibodies and the plasma microneutralization (MN) values. (**I**) Microneutralization results between the 1st and 2nd timepoint measured in 11 ICU patients. (**J**) A correlation between RBD-specific IgG and IgA levels. (**K**) Peak levels of IgA antibodies to RBD in ICU and non-ICU patients. (**L**) The time in days of the seroconversion of RBD IgA antibodies since the start of the symptoms to the cutoff level in ICU and non-ICU patients. (**M**) The trajectory of ICU and non-ICU individual IgA RBD antibody levels plotted over the duration of the disease. The antibody levels in panels K and M are shown as fold changes (FC) as in panels A-D.

We used the LIPS method^24^ to analyze antibody responses to S1, S2 (two subunits of spike protein), RBD, and N protein, which we previously showed to correlate well with the ELISA method as reported earlier^23^. All ICU and non-ICU patients developed IgG antibodies to S1, S2, N and RBD proteins during their hospitalization (Fig. 1A–D). The seroconversion of the COVID-19 patients occurred 12 to 15 days after symptom onset, with an average of 13 days from the start of the disease (medians 15, 13, 12 and 13 days for S1, S2, RBD IgG and N, respectively; Fig. 1E), although overall there was considerable variation in seroconversion kinetics, ranging from 5 to 25 days. Anti-RBD antibodies tended to appear earlier than other antibodies, although without significant difference. We found no significant difference in seroconversion times between non-ICU and ICU cohorts but noted several early antibody responders among non-ICU patients, but none among the ICU patients (Fig. 1F). Following seroconversion, the antibody levels to the four proteins analyzed peaked at around 18–22 days. Although individual antibody levels varied considerably, the non-ICU patients tended to reach peak levels earlier, whereas the ICU patients peaked later but at higher levels, an observation that needs to be viewed in the context of the greater time period over which plasma from the ICU cohort could be sampled (Fig. 1G).

We found high anti-RBD levels among the patients, suggesting strong virus-neutralizing immune responses; we therefore tested 15 ICU plasma samples for their capacity to neutralize SARS-CoV-2 virus and correlated the titration results to LIPS values measuring antibodies to S1, S2, RBD and N proteins. Thirteen of those ICU patients developed a neutralization titer of 1:640, 4 of whom had titers of > 1280. Microneutralization titers correlated well with IgG RBD (ρ = 0.7) and S1 (ρ = 0.73) (Fig. 1H), as reported earlier^19^, and increased over time in 9 of 11 (82%) patients analyzed (Fig. 1I).

All patients, for whom we had plasma samples for a longer period than the first 11 days, developed systemic anti-RBD IgA antibodies, albeit to varying levels, and those antibodies correlated modestly among non-ICU patients with IgG anti-RBD antibodies (Fig. 1J), and with virus neutralization (Fig. 1H). There was no significant difference between ICU and non-ICU groups (Fig. 1K), although the ICU group tended to have more patients with higher levels of RBD IgA antibodies (6 out of 11 ICU *versus* 3 out of 18 non-ICU patients for which IgA antibody data were available), and stronger correlations with RBD IgG antibodies (Fig. 1J,H). The seroconversion of IgA RBD antibodies occurred at the same time of anti-IgG RBD (median 12 days), and tended to be earlier in the non-ICU cohort (median 10 vs 13 days) (Fig. 1L), albeit that the ICU cohort developed higher anti-RBD IgA levels (Fig. 1M).

We also studied the activation of the classical complement pathway in COVID-19 patients by measuring two complement components, C1q and terminal complement complex (TCC). Although COVID-19 patients in general tended to have higher levels of C1q, this difference was not significant (Supplementary Fig. 2A), and there was no difference in TCC levels (Supplementary Fig. 2B).

### Early stage inflammatory mediators

**Figure 2:**
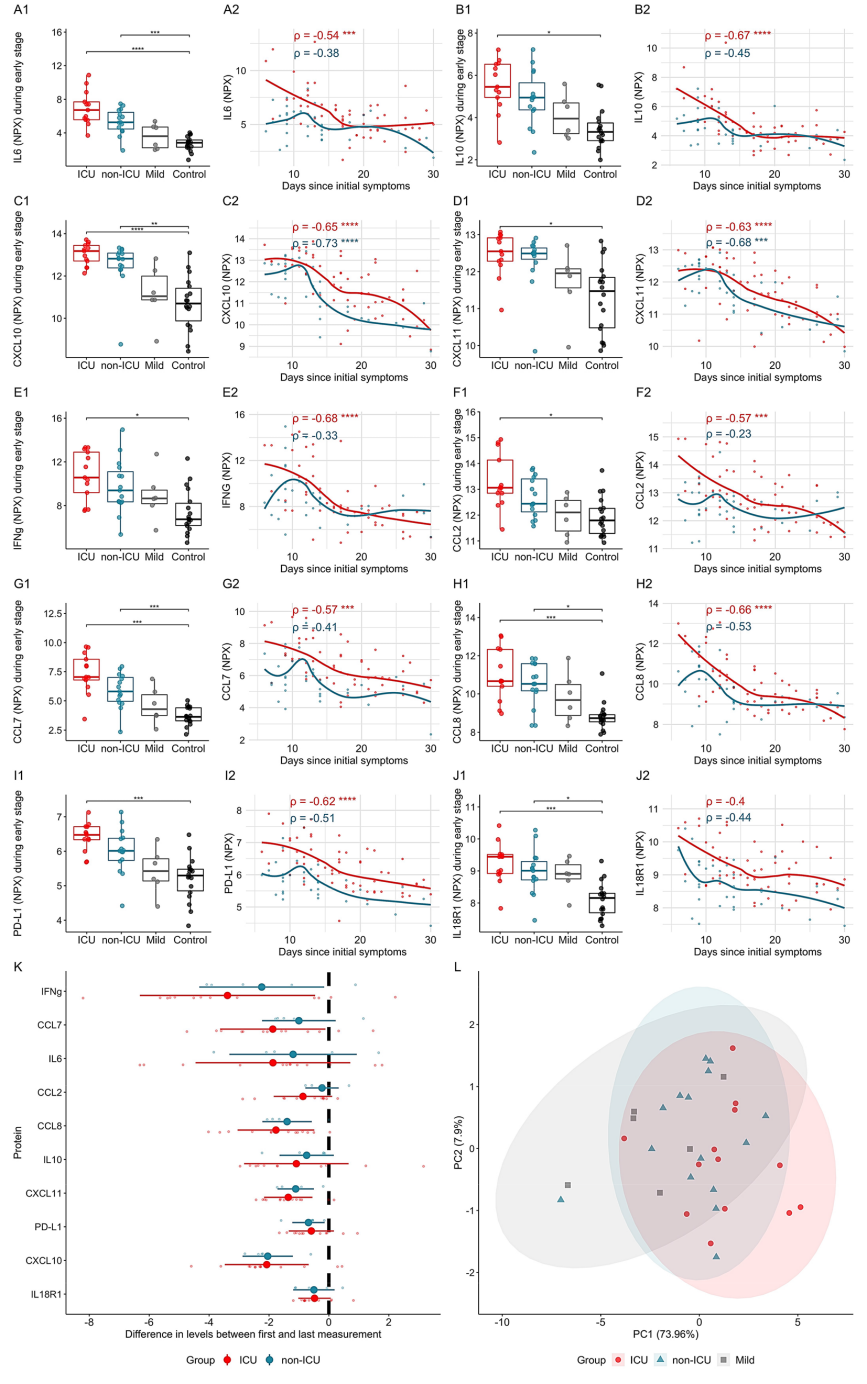
Early disease stage inflammation markers in COVID-19 patients. Inflammatory markers activated at the early stage of the disease and their subsequent decline. (**A1**–**J2**) Group-wise comparisons of at the early stage of the diseases (up to first 3 days) OLINK NPX values (1) and scatterplots (2) where x-axis denotes time in days from the start of the symptoms, y-axis corresponds to OLINK assay’s measurement’s level and the group specific lines are calculated via loess regression. As the early stage samples were not possible to get from all patients, the number of patients studied in box plots is smaller than in scatter plots. (**K**) The difference in OLINK NPX levels between the first and the last measurement of each individual, the proteins on y-axis are sorted by the average difference between ICU and non-ICU groups in descending order. (**L**) PCA plot calculated based on 10 early stage markers.

We applied Olink and Legendplex technologies to undertake a targeted proteomic analysis of individual soluble inflammation-associated proteins and their corresponding correlations in COVID-19 patients. The Olink proximity extension assay (PEA) revealed a significant change of inflammation-associated plasma markers when comparing ICU and non-ICU groups with SARS-CoV-2 negative controls. In ICU patients, we observed increased levels of a triad of IL-6, CXCL10 (also known as IP10) and IL-10. Levels peaked within 24–72 h after hospitalization (i.e. ~ 10 days after initial symptom onset), but declined thereafter before patient dispatch, with the possible exception of IL-6 which remained high in several ICU patients after an initial decrease (Fig. 2A–C). As was recently noted^4^, the IL-6—CXCL10—IL-10 triad showed correlations with disease severity and the levels of these analytes were elevated in the great majority of hospitalized COVID-19 patients, attesting to a highly inflammatory response to the virus.

Other prominent inflammation-associated markers, upregulated at the early stage of disease in ICU patients, were CXCL11, CCL2, CCL7, CCL8, PD-L1, and IL-18R1 (Fig. 2D–J). IFNγ, which is an activator of CXCL10, CXCL11, CXCL9, and other interferon-stimulated genes, was also elevated in the early stages of disease and declined over time, but the analysis of IFNγ was complicated by considerable inter-individual variation among healthy controls (Fig. 2E,K). Nonetheless, while hospital admission levels of the cited cytokines and chemokines were highly variable, there was a constancy to their decline in both patient cohorts, particularly for IFNγ, CCL7, IL-6 and CXCL10 (Fig. 2K). However, the PCA plot with the 10 markers measured at the early stage of the disease did not segregate ICU, non-ICU and the mild disease cohort groups (Fig. 2L). Of note, this group of cytokines and chemokines collectively composes a set of well-established inflammation markers associated with activated myeloid and T cells, that may be highly germane to reports of myeloid and T cell dysregulation in COVID-19.

### Sustained markers related to apoptosis and inflammation

**Figure 3:**
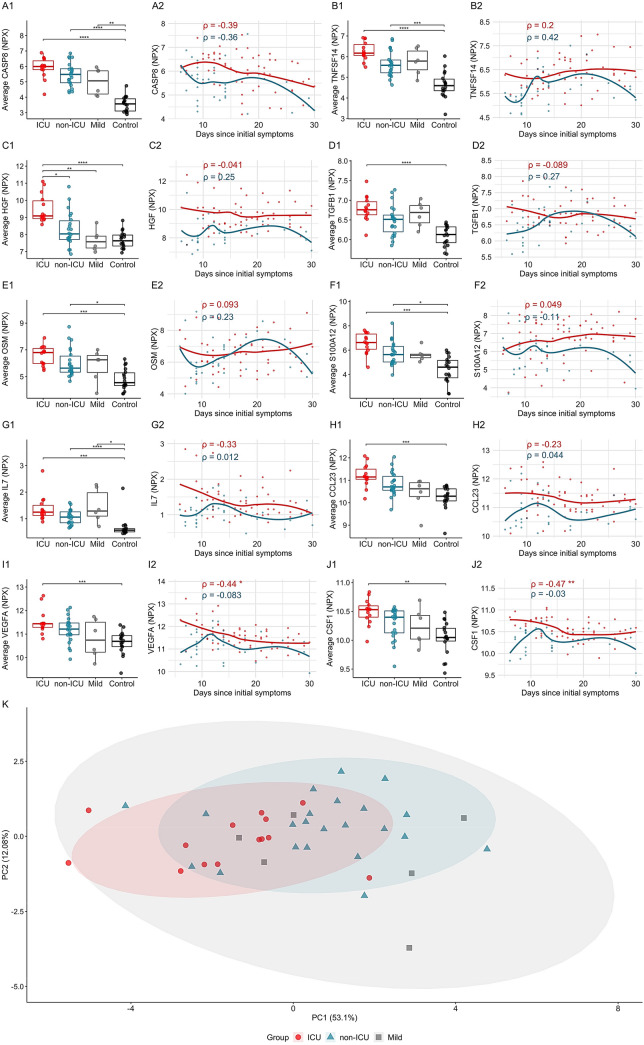
Consistently increased markers of apoptosis and tissue-inflammation in COVID-19 patients. Examples of 10 proteins, including markers associated with apoptotic mechanisms, with increased levels in ICU patients over the disease course. (**A1**–**J2**) PEA profiling NPX values per individual (1) and scatterplots (2) where x-axis denotes time in days since the start of the symptoms, y-axis corresponds to the measurement’s level and the group specific lines are calculated via loess regression. (**K**) PCA plot calculated based on 10 markers' average levels consistently upregulated in COVID-19.

Possibly in relation to the overt subset-selective T-cell apoptosis reported for COVID-19^25^, we found in ICU patients significantly increased and sustained levels of plasma markers associated with apoptosis. Among these, we observed consistent increases in the levels of CASP8 and TNFSF14 (Fig. 3A,B, Supplementary Table 1), two biomarkers that are related to inflammation combined with apoptosis. Their levels were significantly greater among ICU and non-ICU cohorts compared to healthy controls, and were sustained over the disease course. Interestingly, the third top upregulated marker HGF was specific to and significantly higher among ICU patients when compared to all other groups (Fig. 3C, Supplementary Table 1), and the capacity of HGF to largely discriminate ICU from non-ICU patients was observed over the complete time-course of sampling (Fig. 3C). HGF is best characterized in relation to anti-apoptotic function, possibly reflecting a negative feedback mechanism.

Additional plasma proteins with similar patterns included markers such as TGFB1, Oncostatin M (OSM), S100A12, IL-7, CCL23, VEGFA and CSF-1 (Fig. 3D–J). Notwithstanding some very considerable overlap, a PCA of the average values of the 10 cited markers seemed more efficient at segregating the ICU and non-ICU cohorts (Fig. 3K) than did the collection of cytokines and chemokines activated at early disease (Fig. 2L). This may reflect their different patterns of regulation in COVID-19, and argues for the measurement of apoptosis-related proteins in other, independent cohorts.

To validate the PEA (Olink) profiling, we compared ICU, non-ICU and mild-disease patients by Legendplex assay. This confirmed severity-associated increases levels of the triad of IL-6, CXCL10, and IL-10, and provided some evidence of TNF upregulation (Supplementary Fig. 3A–M). Moreover, the analysis also confirmed the decline of IL-6, CXCL10, IL-10 and IFNγ cytokine levels in both the ICU and non-ICU cohorts over time, and the lack of convincing segregation in PCA plot (Supplementary Fig. 3N,O). In short, the results of the two plasma protein screening platforms cross-validated (Supplementary Fig. 4). We further tested 2 soluble proteins, sCD25 and sCD14, that reflect activated immune cells and are commonly upregulated in sepsis. Indeed, seemingly reflective of dynamic immune responses, we found increased peak levels of sCD25, shed by T cells, and sCD14, shed by macrophages and monocytes in ICU and non-ICU patients (Supplementary Fig. 5A,B), which clustered together but not with early-stage inflammatory markers (Supplementary Fig. 5C).

### Discrete immune marker clusters segregate COVID-19 patients

**Figure 4:**
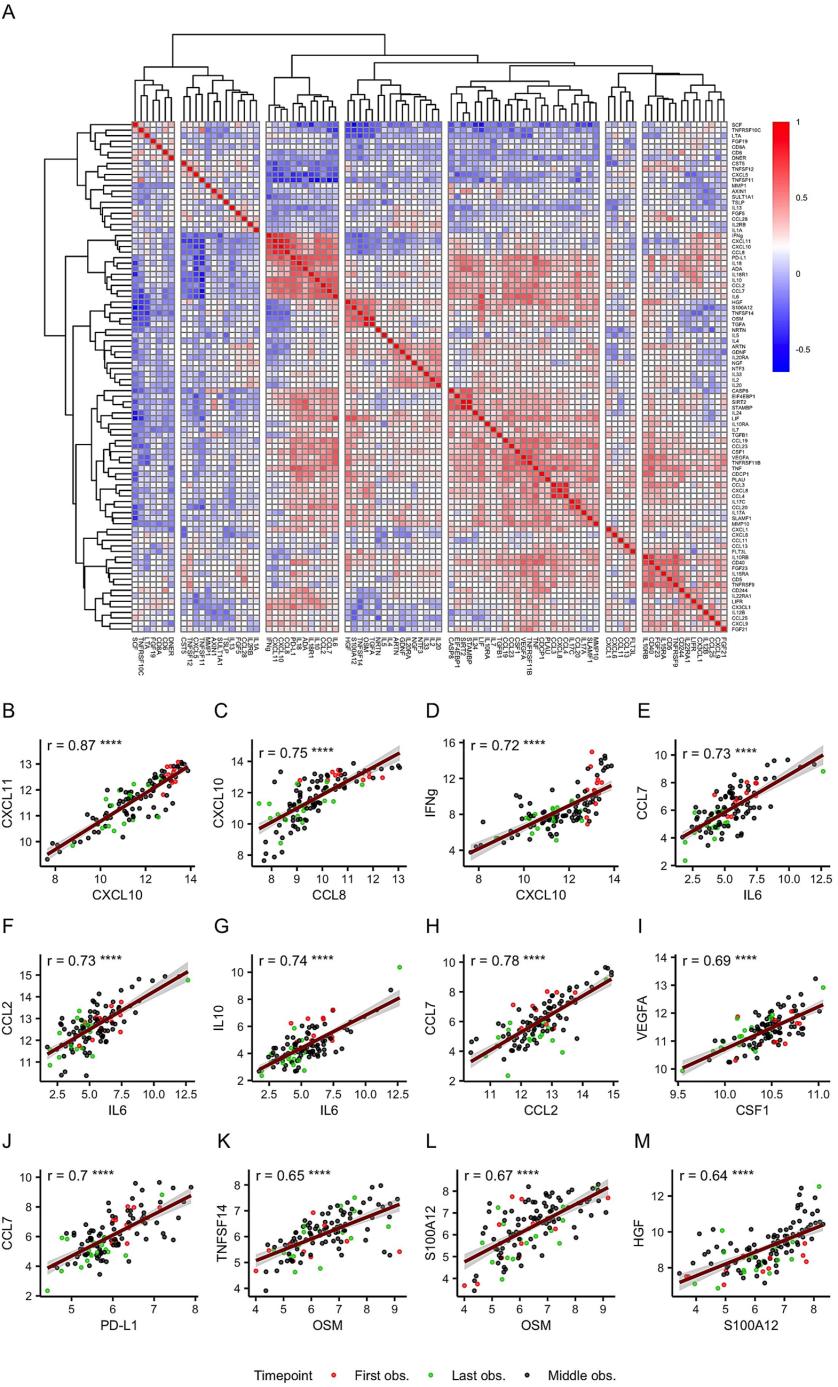
Cluster analysis of plasma protein correlations values. (**A**) The heatmap of seven distinct clusters based on Spearman’s correlation matrix from all PEA analysis NPX measurements based on hospitalized patient data*.* (**B**–**M**) Scatterplots of pairwise Pearson correlations of selected PEA measurements. Linear regression line shows overall trend between two measurements and individual observations are colored according to the sampled timepoints; at the hospitalization (first observation, red), before leaving the hospital or passing away (last observation, green) or between those aforementioned timepoints (middle observation, black).

Since the proteomic analysis of secreted proteins suggested distinct patterns of regulation, we performed a cluster analysis of all PEA (Olink) profiling markers that were altered in ICU and non-ICU patients (Fig. 4A). We found 7 clusters of plasma proteins that segregated based on Spearman correlations. As anticipated, the clustering analysis confirmed a strong correlation between early-stage inflammatory markers, forming a discrete profile of IL-6, IL-10, CXCL10, CXCL11, IFNγ, CCL2, CCL7, CCL8, IL-18, IL-18R1 and PD-L1. We also observed a cluster containing HGF, TNFSF14, S100A12, OSM and TGFA. In contrast, CASP8 clustered separately, together with EIF4EBP1, SIRT2 and STAMBP and with TGFB1 and IL-7. Strikingly, TNFSF11 (TRANCE) and CXCL5 showed a strong negative correlation with most of the early-stage inflammatory markers indicating an opposite trend of these markers in COVID-19 pathogenesis.

The correlations were disease-associated as they were different in healthy controls. Moreover, the samples collected at the beginning of hospitalization manifested a substantially greater degree of systemic inflammation, exemplified by the strong correlation between CXCL10, CXCL11, IFNγ and PD-L1 when compared to samples collected during the disease course or immediately prior to discharge (Fig. 4B–M, Supplementary Table 2). This suggests the potential of admission levels of certain analytes to be prognostic of subsequent disease course, as has been recently proposed^4,5^. Such risk-based patient stratification might be further improved by assessments of markers with strong disease-associated correlations, e.g. between HGF and TNFSF14, and between IL-6 with CASP8 and HGF (Fig. 4H,J,M). Indeed, some of the strong correlations reflect associations between related family members mediating immune-stromal crosstalk such as that between IL-6 and LIF (Fig. 4E), and that between IL-6 and OSM.

### Distinct immunotype in ICU patients

**Figure 5:**
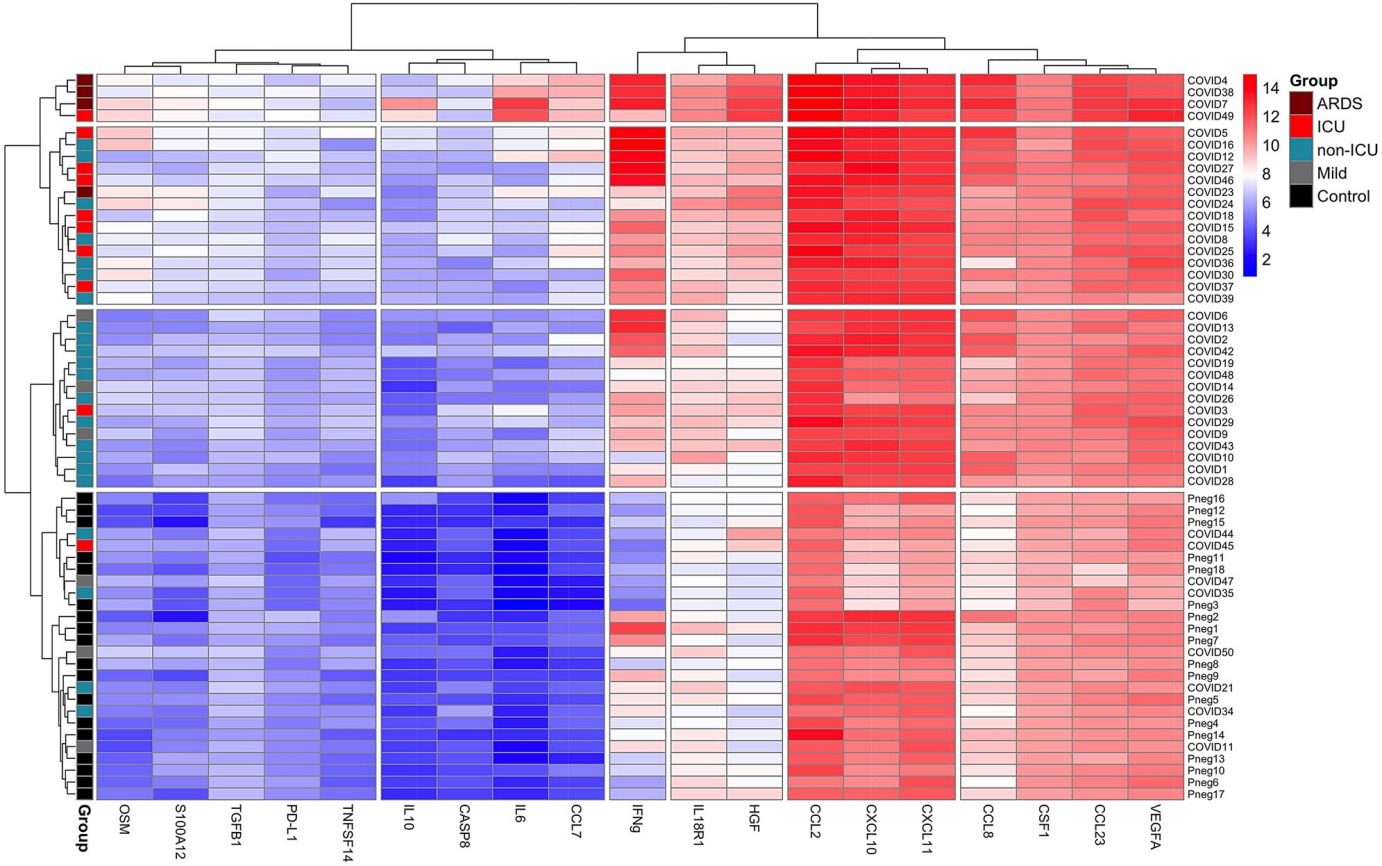
Plasma marker analysis reveals distinct clusters in COVID-19 patients. Cluster analysis of maximal levels (NPX) of 19 proteins (the proteins presented in Figs. 2 and 3 except IL7 due to low overall levels) in 60 individuals (14 ICU-s of which 4 developed ARDS, 22 non-ICU-s, 6 mild cases and 18 controls). The analysis identified 4 clusters of individuals and starting from the top, the first cluster included ICU patients with ARDS and 1 patient (COVID49) who died before receiving ARDS diagnosis. The second cluster contained 16 (1 ARDS, 7 ICU and 7 non-ICU) patients. Cluster three included mostly non-ICUs and cluster four mostly controls.

The patients with severe disease displayed stronger upregulation of discrete inflammation and apoptotic markers suggesting distinct immunotypes that might relate to disease characteristics and outcomes. To determine whether patients with severe COVID-19 could be categorized based on their expression of secreted proteins, we conducted clustering analysis using patients’ marker measurements at their peak levels. At least 4 disease subgroup clusters emerged (Fig. 5). The two top patient clusters, including most of the ICU patients were represented by the strongest activation of multiple cytokines and chemokines, including OSM and S100A12. Strikingly, the most prominent distinct immunotype segregated by HGF, IL6 and CCL7 and was represented by three patients diagnosed with ARDS (COVID4, COVID7, and COVID38) and one patient who died (COVID49). The presence of ICU-specific immunotypes may usefully reflect accelerated inflammatory responses and pathophysiological mechanisms in subgroups of severe patients, which may require tailored immunomodulatory treatment strategies.

## Discussion

We have undertaken a longitudinal comparison of antibodies and plasma proteins, altogether comprising 101 different markers, in COVID-19 ICU and non-ICU patients and have contextualized them with patients with mild disease and age-matched negative controls. This has revealed a signature of hyperinflammation apparent early in the disease-course, long before seroconversion, for both ICU and non-ICU patients. However, rather than being chaotic, the signature comprised elevated levels of discrete cytokines and chemokines, some of which correlated with disease severity. Moreover, those cytokines and chemokines target myelomonocytic cells and T cells that have been reported to display the most overt immunophenotypes in COVID-19. Those phenotypes include severe, subset-selective T cytopenia, possibly in relation to which, a set of proteins associated with apoptosis form a cardinal feature of COVID-19.

As previously reported, we found high levels of acute-phase inflammatory cytokine IL-6, targeted by tocilizumab in COVID-19 patient treatments. CD4^+^ T cells and monocytes are considered as the primary sources of high IL-6 levels in COVID-19, which in turn correlate with decreased monocytic HLA-DR expression, one of the hallmarks of the disease^9,26^. Furthermore, increased levels of other pro-inflammatory cytokines and chemokines, including CXCL10, CXCL11, and IL-10, were already characteristic to patients upon admission to hospital^15^. Accordingly, increased albeit variable levels of IFNγ were present during the early stages of hospitalization.

In parallel, the monocyte-attracting chemokines CCL2, CCL8, and CCL7, associated with septic shock and tuberculosis, were elevated supporting the role of monocytes in disease pathogenesis. Indeed, the analysis of bronchoalveolar lavage fluid from the patients detected a substantial increase in lung macrophages with enrichment of monocyte-derived FCN1^+^ macrophages^27,28^. Interestingly, transgenic mice expressing the human ACE2 gene and infected with SARS-CoV-2 also demonstrated an accumulation of monocytes and macrophages in the lungs after infection, providing evidence that SARS-CoV-2 precipitates monocyte influx and macrophage accumulation during infection^29^. Moreover, we detected increased levels of soluble sCD14 produced by myeloid cells and has hitherto been correlated with the severity of bacterial sepsis or septic shock.

However, the most conspicuous, sustained differences observed over the disease course were in CASP8, HGF, TNFSF14, and in TGFB1, that are each associated with apoptotic mechanisms and inflammation. They share several mechanistic features and have multiple implications in cardiovascular and other chronic inflammatory diseases. CASP8 is released to the extracellular medium after the apoptotic stimulation of myeloid and T cells. TNFSF14 is primarily produced by activated T cells and myeloid cells, is increased in plasma from patients with acute ischemic atherosclerotic stroke^30^, and is involved in the progression of chronic heart failure^31^. TNFSF14 was recently reported to be upregulated in COVID-19 patients together with OSM and S100A12^6^, which both showed higher levels in our analysis. HGF, whose upregulation was specific for ICU group, has anti-apoptotic effects, and its levels are elevated in chronic inflammatory diseases. HGF frequently counteracts TGFB1, another cytokine involved in apoptosis, and expressed at elevated levels in COVID-19 patients^32^. Our finding is consistent with other recent studies showing the association of HGF with COVID-19 severity^6,33^. It might therefore be considered that the apoptotic events that drive increased HGF levels, underpin T cell apoptosis as a fundamental trait that contributes to SARS-CoV-2-specific immune pathology; hence, the association of HGF with disease severity and ICU requirement.

Clustering all plasma markers indicated segregation between early-stage markers (IL-6, CXCL10, CXCL11, CCL2, CCL7, CCL8, IL-10 and IFNγ) and sustained markers of apoptosis and inflammation (HGF, TNFSF14, OSM and S100A12). Their differential role in COVID-19 pathogenesis remains to be studied, however it is conceivable that the early stage markers are related to initial strong inflammatory response to overwhelming viral infection and are driven by monocyte-macrophage activation whereas the sustained pattern of several apoptosis-related markers is related to T-cell apoptosis and hyperinflammation-induced tissue destruction^12,25^. The cluster analysis of the patients based on 19 selected markers showed clear separation of severe cases including 3 ARDS-diagnosed cases and one patient who died, who all had higher levels of IL-6, HGF and CCL7 during the disease compared to other ICU patients. In addition to these three markers, the ICU group tend to be separated from non-ICU patients by OSM and S100A12, whereas all COVID-19 patients can be distinguished from healthy controls by CASP8, IFNγ, IL-18R and CCL8. It would be important to follow the dynamics of plasma markers in larger cohorts as well as in recovered patients after the infection, and to study non-infected elderly individuals to decipher whether the higher pre-infection levels of the identified markers might pose risk for the development of severe COVID-19 disease.

In sum, the findings of our study can inform our understanding and management of COVID-19. The proteomic analysis of inflammation-associated plasma markers will enable better patient stratification and early identification of those with severe disease. While many major unknowns remain to be tackled, this study has highlighted the potential utility of tracking, and potentially targeting, a discrete spectrum of inflammatory mediators and apoptosis regulators.

## Materials and methods

### Samples

Local Ethical Committee (The Ethical Committee at the University of Tartu) approval (#312/M-1) was received for the studies. The informed consent was obtained from SARS-CoV-2 negative control donors used in the study. For COVID-19 patients, a waiver of consent was granted for obtaining excess clinical laboratory blood. The samples were collected in April–May, 2020, during the country’s situation of emergency and informed consent of participating subjects was waived by the Ethical Committee at the University of Tartu. This was related to the COVID-19 situation in Estonia and was based on the exception in Estonian Law on Personal Data, which gave the right for the exception to obtain informed consent in life-threatening emergencies, in case of situation with overwhelming public interest and on a condition that the waiver will not adversely affect the rights and welfare of the subjects. Plasma samples were obtained from 40 COVID-19 patients (Table 1) hospitalized at the Tartu University Hospital, Estonia. Fifteen patients were treated in ICU and 25 in ordinary COVID-19 ward. Most of the ICU patients required mechanical ventilation within the first 10 days after the onset of symptoms with a median duration of the intensive care unit (ICU) stay of 10 days (range 1–54 days). Moderate COVID-19 patients were hospitalized for a median duration of 11 days (range 1–44 days). The diagnoses for all patients were confirmed by PCR analysis for SARS-CoV-2 virus. During their hospitalization in April–May 2020, some patients were treated with hydroxychloroquine but not with remdesivir, dexamethasone or IL-6 inhibitors. The patients nevertheless, had several comorbidities and were given corresponding treatments. The samples from a group of 6 patients with mild disease were collected from the individuals who came to emergency medical unit, were tested positive for SARS-CoV-2 but sent home for quarantine and not admitted to the hospital. We studied altogether 119 healthy controls (age range 23–87 years) without recent infection or COVID-19 symptoms (fever or cough) for last month. Blood samples of two groups of patients (mostly over 60 years of age) without COVID-19 disease were collected from Internal Medicine unit and CRP under 5 mg/L: one group of 18 individuals was studied for clinical blood analysis and another group of 18 controls for the expression of multiple plasma proteins. In addition, 70 control individuals, including healthy blood donors, were studied for antibody reactivities.

### LIPS

SARS-CoV-2 S (S1 aa 1–680 and S2 aa 820–1211), S1 RBD (aa 329–538), and N (aa 2–419) fragments were cloned into pNanoLuc vector, and LIPS was performed as reported^23^. The HEK293 cell lysates containing NanoLuc‐fusion proteins (0.5—1 × 10^6^ luminescence units; LU) were incubated with plasma samples and Protein G Sepharose beads (Creative BioMart) to capture antibodies (in 1:40 dilution). After washing, substrate was added (Nano-Glo™ Luciferase Substrate, Promega), and luminescence was measured in VICTOR X Reader (PerkinElmer Life Sciences). Results are expressed as fold changes (FC) of LU-s (FC = LU sample/average LU of 5 healthy control samples, the discrimination level is mean plus 2 standard deviations of control samples).

### Microneutralization

The assay was performed in a BSL-3 laboratory as described previously^18^. Briefly, fifty plaque-forming units (PFU) of the SARS-CoV-2/Finland/1/2020 strain, passaged in Vero E6 cells, were added to the plasma dilutions and incubated for 1 h at 37 °C. Neutralizing antibody levels were assessed by cytopathic effect (CPE) stained with crystal violet. The neutralization endpoint titer was determined as the endpoint of the serum that inhibited the SARS-CoV-2 infection in parallel wells. The titer ≥ 20 was considered as positive.

### ELISA

Quantification of human C1q, terminal complement complex (TCC), human Interleukin 2 receptor alpha (CD25/IL-2 Rα) and soluble Presepsin (sCD14) were performed by commercial ELISA kits: C1q (catalogue number HK356-02, Hycult Biotech, Netherlands), TCC (product code COMPL TCC RUO, SVAR, Sweden), CD25/IL-2 Rα (catalogue number DY223, R&D Systems, USA) and sCD14 (catalogue number abx576557, Abbexa, UK). All ELISAs were performed as indicated by the manufacturer and optical density was measured with Multiscan MCC/340 ELISA reader (Labsystems, USA).

### LEGENDplex profiling

Bead-based LEGENDplex™ Human Anti-Virus Response Panel 13-plex kit (catalogue number 740349, BioLegend, USA) was used to quantify 13 human proteins, including interferons (α, β, γ, λ1 and λ2/3), interleukins (1β, 6, 8, 10, 12), TNF-α, IP-10 and GM-CSF. Assay was performed according to manufacturer’s protocol. Data were acquired by BD LSRFortessa™ flow cytometer (BD Biosciences, USA) and LEGENDplex™ Data Analysis Software was used for data analysis (BioLegend).

### Olink proximity extension profiling

In total, 92 inflammation-related protein biomarkers were measured in plasma by the Proximity Extension Assay technique by using the Proseek Multiplex Inflammation panel by Olink^®^ Proteomics. The assay uses two oligonucleotide-conjugated antibodies that bind to protein targets. Upon binding to the protein epitope, the paired oligonucleotide sequences are amplified through a quantitative real-time PCR (qRT-PCR) reaction. Data is then generated using normalized protein expression (NPX) values on a log2 scale whereby a higher NPX correlates with higher protein expression. Proteins containing NPX values > 50% below the assay’s limit of detection (LOD) were excluded from the analysis. The data were pre-processed by Olink^®^ using NPX Manager software.

### Statistical analysis

Statistical analysis was performed in R (version 4.0.2). The units of measurements taken for given analysis are as follows. Firstly, CRP was measured in mg/L and PCT in µg/L. Microneutralization titer is expressed in dilutions. The levels of antibodies are expressed as fold changes based on LIPS measurements (LU): antibody FC = LU sample / mean (LU of 5 healthy control samples). Olink’s protein levels are represented as normalized protein expression (NPX) units, which are inherently in Log2 scale then Log2 was also taken from Legendplex values to keep the similar range. Statistical significance was determined using two-sample Wilcoxon test (also known as Mann–Whitney test). The significance symbols on boxplots are: * = adj. P ≤ 0.05, ** = adj. P ≤ 0.01, *** = adj. P ≤ 0.001 and **** = adj. P ≤ 0.0001. Bonferroni correction was used for all statistical and correlation tests. In addition, because OLINK contains many highly correlated proteins, the number of independent components from PCA that explained 99% of variance was obtained and subsequently used for adjusting p-values. Correlation analysis was done using Spearman’s rank correlation except for Figs. 1J and 4B–M were Pearson correlation was used. Local regression was used to express relationship between variables on all figures except in Figs. 1J and 4B–M were linear regression was used instead. Cluster analysis based on Euclidean distance and subsequent visualizations as heatmaps were done using R package pheatmap^34^.

## Supplementary information

Supplementary Information 1.

Supplementary Information 2.

Supplementary Information 3.
