# Supplementary material for: Longitudinal proteomic profiling reveals increased early inflammation and sustained apoptosis proteins in severe COVID-19
Source: Sci Rep. 2020 Nov 25;10:20533. doi: 10.1038/s41598-020-77525-w (PMC7689507; doi:10.1038/s41598-020-77525-w)

**Supplemental Figures**

**Longitudinal proteomic profiling reveals increased early inflammation and sustained apoptosis proteins in severe COVID-19**

Short title**: Increased apoptotic markers in COVID-19**

Liis Haljasmägi*^1^, Ahto Salumets*^1,2^, Anna Pauliina Rumm*^1^, Meeri Jürgenson^1^, Ekaterina Krassohhina^1^, Anu Remm^1^, Hanna Sein^1^, Lauri Kareinen^3^, Olli Vapalahti^3^, Tarja Sironen^3^, Hedi Peterson^2^, Lili Milani^4^, Anu Tamm^5^, Adrian Hayday^6^, Kai Kisand^1^, Pärt Peterson^1^

**Supplemental Figure Legends**

**Supplementary Figure 1.** **Timeline of sample acquisition and key findings from blood measurement in COVID-19 patients**. The ICU and non-ICU patient groups are shown in red and green, respectively, and healthy controls in black. (A) The time points the patients were sampled for quantifying plasma proteins with either OLINK, Legendplex, or both. The x-axis shows the time from the day of initial symptoms and y-axis shows individual patients. (B1-I2) Blood measurement levels between the three groups in individual patients and controls. The asterisks represent the statistical significance of Wilcoxon rank sum test. (B and C) CRP and PCT levels at the hospitalization (B1 and C1) and all datapoints during the disease (B2 and C2). (D and E) The proportions of lymphocytes and neutrophils at the beginning of the disease (D1 and F1) and all datapoints of lymphocyte (D2) and neutrophil (E2) values. (F-G). NLR and overall WBC counts at the beginning of the disease (F1 and G1) and all datapoint values (F2 and G2). (H-I) The proportions of basophils and eosinophils at the beginning of the disease (H1 and I1) and all datapoint values (H2 and I2).

**Supplemental Figure 2. Complement activation by analysis of C1q levels and terminal complement complex activation in hospitalized patients.** The ICU, non-ICU and control groups were studied for C1q (Hycult Biotech, Netherlands and terminal complement complex (COMPL TCC RUO, SVAR, Sweden) levels by ELISA method according to the manufacturer’s instructions.

**Supplemental Figure 3 (A-O).** Inflammatory markers analyzed by Legendplex 13-plex Inflammation panel between ICU, non-ICU and mild disease cases. The levels of 13 activated cytokines at the early stage of the disease and their corresponding trajectories are shown. (A1-J2) Group-wise comparisons of at the early stage of the diseases (up to first 3 days) (1) and scatterplots (2) where x-axis denotes time in days from the start of the symptoms, y-axis corresponds to cytokine level and the group specific lines are calculated via loess function. (K) The difference in cytokine levels between the first and the last measurement of each individual, the proteins on y-axis are sorted by the average difference between ICU and non-ICU groups in descending order. (L) PCA plot calculated based on analyzed cytokine levels.

**Supplemental Figure 4.** Correlation between Olink PEA assay and Legendplex 13-plex Inflammation panels overlapping markers (CXCL10, IL-10, IL-6 and IL-8).

**Supplemental Figure 5.** Soluble CD25 (A) and CD14 (B) values in patient groups and in controls. (C) Heatmap of correlation matrix of sCD25 and sCD14 with markers in PEA profiling.

**Supplementary Figure 1.**


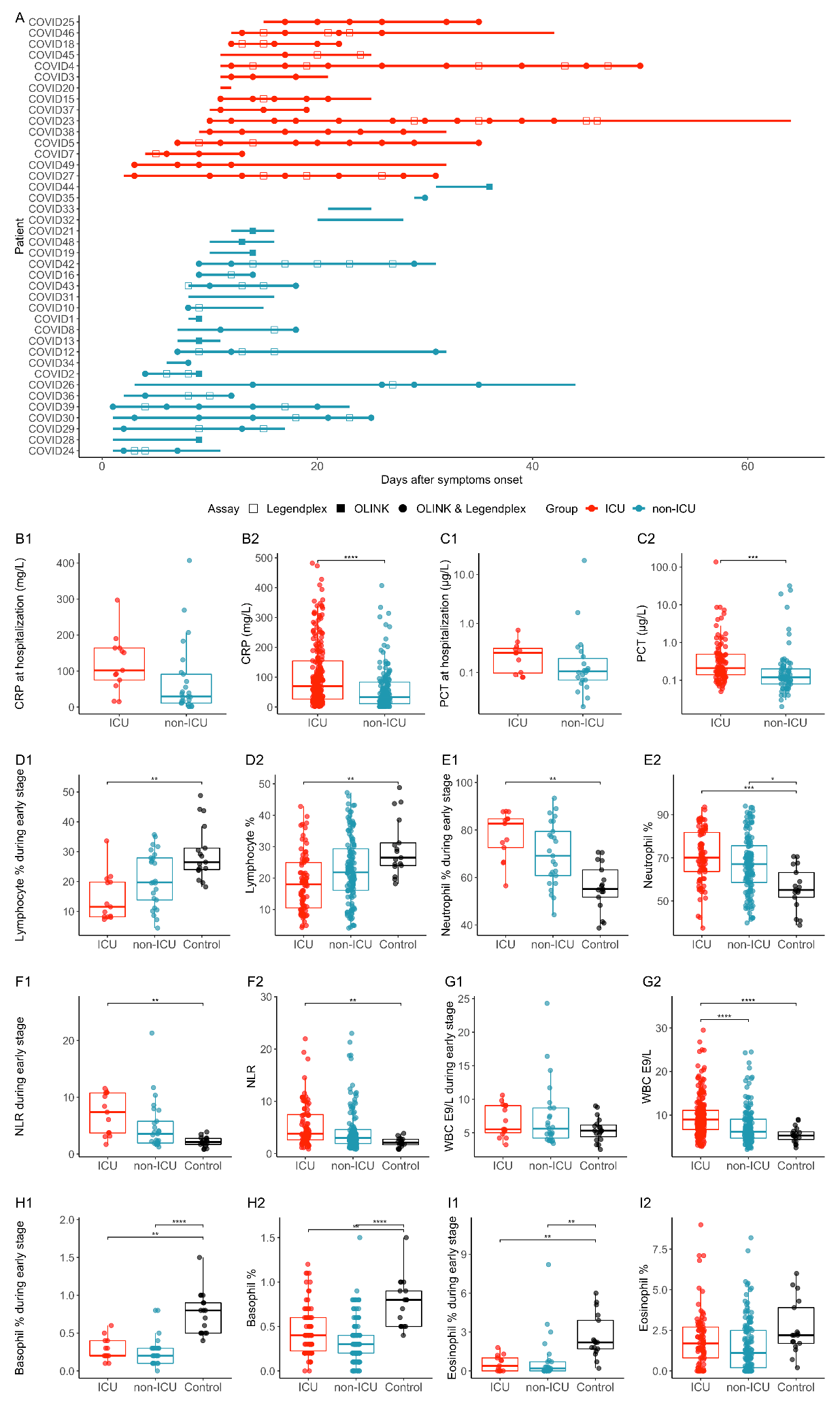


**Supplemental Figure 2.**

**Supplemental Figure 3.**


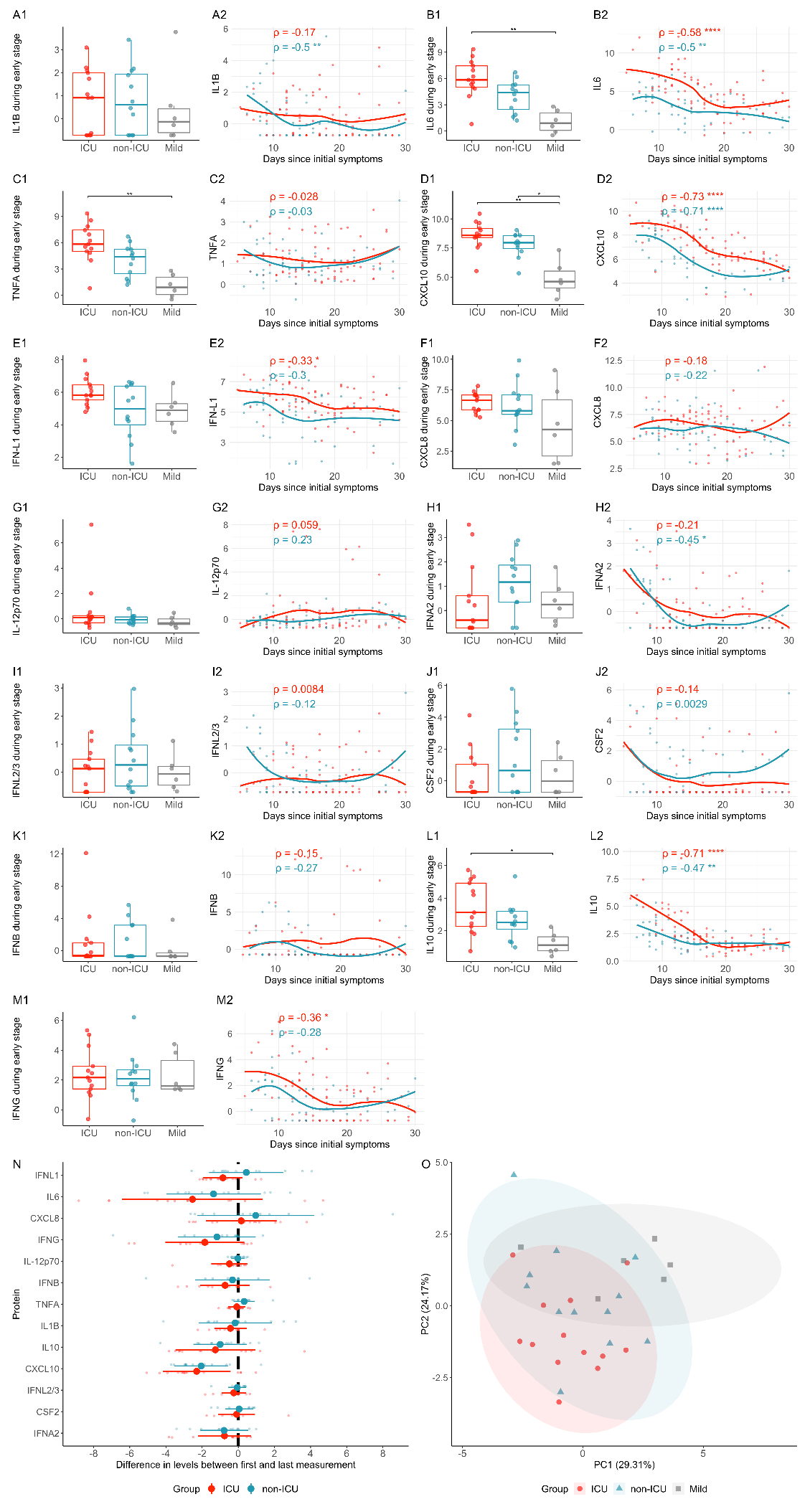


**Supplemental Figure 4.**

**
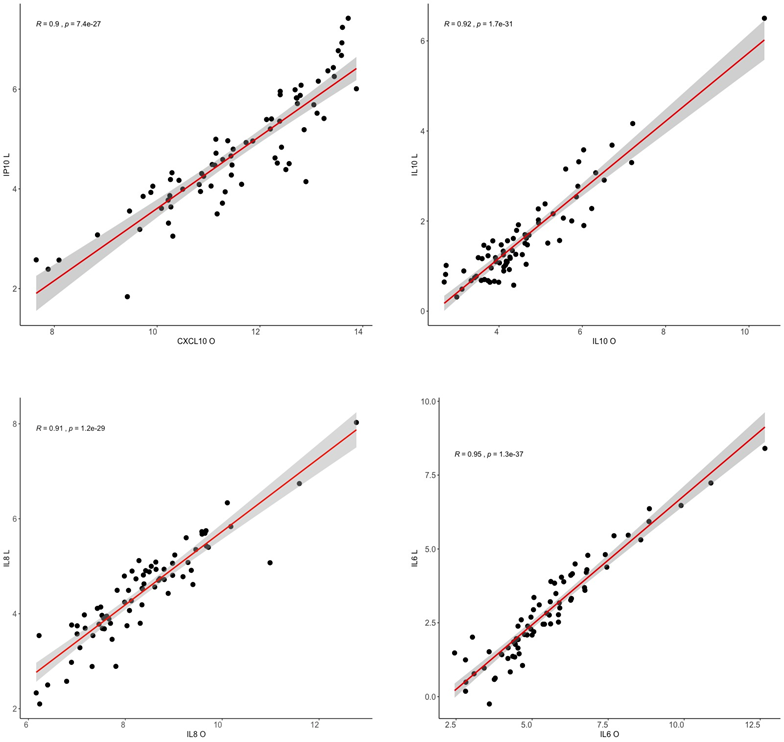
**

**Supplemental Figure 5**.


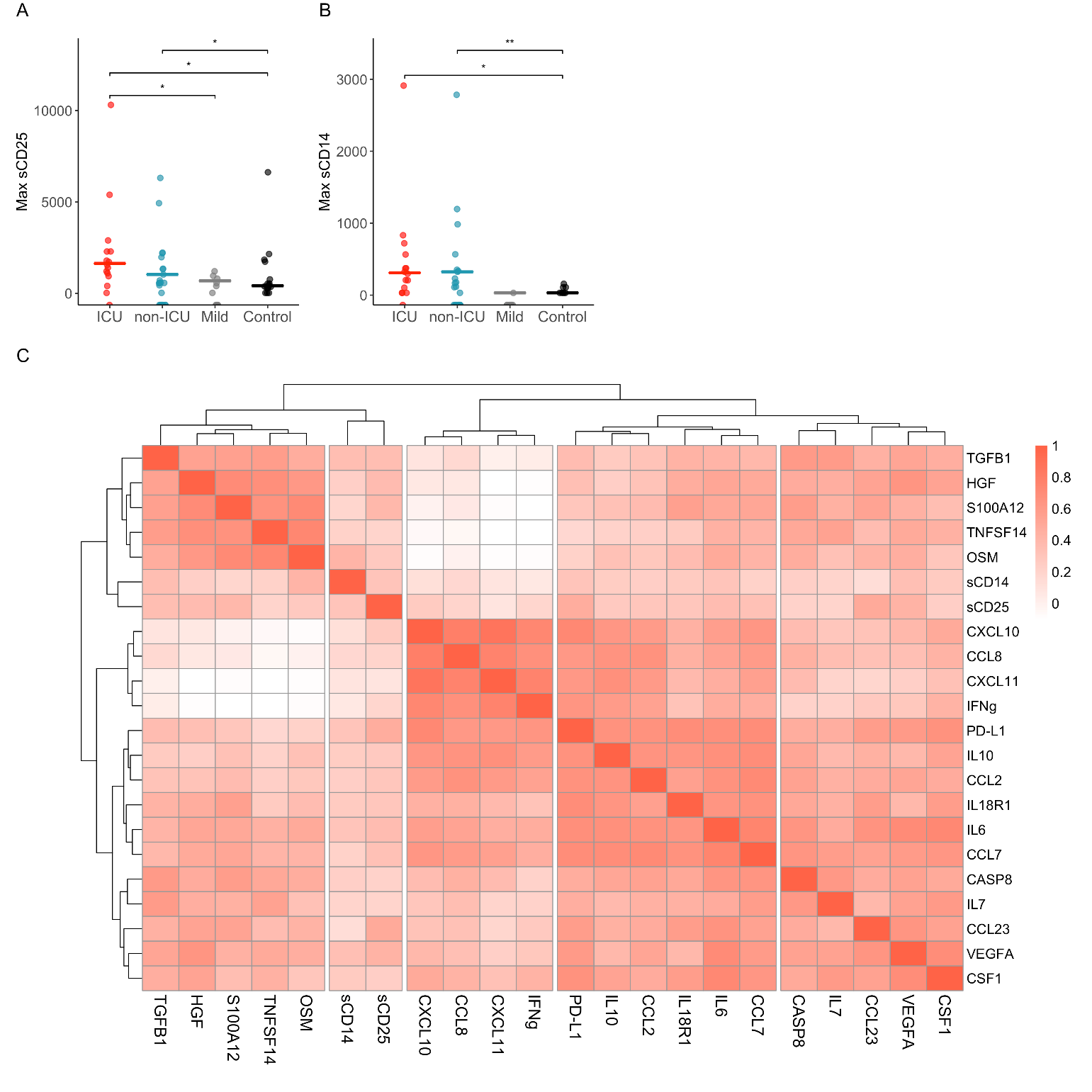

Supplement: Supplementary file 1 — Supplementary Information 1. [file 41598_2020_77525_MOESM1_ESM.docx]
